# First description of *Blastocystis* sp. and *Entamoeba* sp. infecting zoo animals in the Qinghai-Tibetan plateau area, China

**DOI:** 10.3389/fcimb.2023.1212617

**Published:** 2023-06-08

**Authors:** Tongsheng Qi, Wangli Zheng, Liangting Guo, Yali Sun, Jixu Li, Ming Kang

**Affiliations:** ^1^ College of Agriculture and Animal Husbandry, Qinghai University, Xining, China; ^2^ State Key Laboratory of Plateau Ecology and Agriculture, Qinghai University, Xining, China; ^3^ Qinghai Provincial Key Laboratory of Pathogen Diagnosis for Animal Diseases and Green Technical Research for Prevention and Control, Qinghai University, Xining, China

**Keywords:** zoo animals, *Blastocystis* sp., *Entamoeba* sp., wildlife park, Qinghai-Tibetan plateau area

## Abstract

Protozoan parasites are a well-known threat to human health, particularly for people working at or visiting zoos, and potentially cause zoonotic diseases in humans. Captive wildlife may be potential reservoirs for human infection with protozoan parasites. Therefore, focusing on zoonotic protozoan infections in zoo animals is critical. However, there is no report on this topic in the Qinghai-Tibetan Plateau region. In this study, a total of 167 and 103 fecal samples were collected from 12 animal species from Qinghai-Tibet Plateau Wildlife Park in winter and summer, respectively, to detection the prevalence of infections and subtype distribution with *Entamoeba* sp., *Cryptosporidium* sp., *Giardia duodenalis*, *Enteromicrosporidia bieneusi* sp., *Blastocystis* sp. by PCR assay. The results showed that a total of 21 fecal samples collected in winter, including from 2 white-lipped deer, 8 Sika deer, 6 blue sheep, 2 wolves and 3 bears, were positive for *Entamoeba*, with a 12.6% (21/167) positive rate. However, 4.9% (5/103) of animals in summer were positive for *Entamoeba*, including 1 snow leopard, 1 tiger, 1 Tibetan argali and 2 mouflon. Moreover, 1 white-lipped deer and 1 bear were found to be positive for *Blastocystis* sp., one zoonotic STs (ST10) was identified and found in white-lipped deer. We found no effect on season on *Blastocystis* sp. and *Entamoeba* sp. colonization. To the best of our knowledge, this study is the first description of *Blastocystis* sp. and *Entamoeba* sp. infecting zoo animals in the plateau area. The findings provide the latest data on *Entamoeba* sp. and *Blastocystis* sp. in zoo animals in China.

## Introduction

Wildlife has been suggested to play important roles in the ecology and transmission of emerging animal infectious diseases (Miller et al., 2013). An increasing number of studies have found that diseases caused by parasites seriously harm wild animals. The pathological process of animal parasitic diseases is slow and can lead to anemia and emaciation, causing mechanical damage to various tissues and organs, and leading to death. Due to the slow pathological process, parasitic diseases are often covered by some non-infectious diseases or nutritional deficiency diseases, becoming the source of infection and causing greater harm. Infectious diseases caused by protozoan parasites are common in zoo animals worldwide and may represent a risk to human health, particularly for people working at or visiting zoos (Delport et al., 2014). The public health significance of these enteric protists depends on the distribution of genotypes and/or subtypes. The Qinghai-Tibet Plateau Area (QTPA), the largest plateau with the highest average altitude on the planet, is located in northwestern China. Due to the specific climate (i.e., low average annual temperature, low rainfall, and changeable climate), a variety of unique animals are maintained on the QTPA, including Przewalski’s gazelle and the white-lipped deer. However, only a few investigations of the presence of protozoan parasites have been reported in zoo animal sources in this area.

*Entamoeba* sp. and *Blastocystis* sp. are two common protozoan pathogens that parasitize the gastrointestinal tract and can infect many animal species, causing widespread epidemics in various countries (Cian et al., 2017; Deng et al., 2019; Ren et al., 2021). As these parasites have a broad host range, they are considered major zoonotic pathogens. In recent years, *Entamoeba* has been found in amphibians and many other hosts, including humans, nonhuman primates, birds, mammals, and reptiles, and is listed as the third most common cause of parasite disease-associated mortality (Stensvold et al., 2010; Ren et al., 2021). The transmission of *Entamoeba* between hosts may occur through oral ingestion of mature cysts or fecal–oral contact. Epidemiological studies have shown that *Blastocystis* sp. infections predominantly occur in immunocompromised individuals and those in close contact with animals (Wawrzyniak et al., 2013). *Blastocystis*, transmitted *via* the fecal–oral route, is a strictly anaerobic protozoan that inhabits the gastrointestinal tract in humans and animals (Matsubayashi et al., 2018). There is supporting evidence that some human infections may be caused by zoonotic transmission of *Blastocystis* sp. (Parkar et al., 2010; Wang et al., 2014). Based on polymorphisms of small subunit (SSU) gene of *Blastocystis* sp., 28 subtypes (STs) consisting of ST1 to ST17, ST21, ST23 to ST29 and ST30-ST32 have been identified in humans and domestic and wild animals worldwide (Chen et al., 2023). *Blastocystis* has also been identified in other animals, including woolly monkeys, dogs, ring-tailed lemurs, ostriches, giraffes, kangaroos, and snow leopards (Li et al., 2020a).

*Cryptosporidium* sp., *Giardia duodenalis*, and *Enteromicrosporidia bieneusi* sp. are important causes of diarrhea (Wang et al., 2018; Ma et al., 2020). They also infect a wide range of animals, including livestock, nonhuman primates, companion animals, and wild animals. (Feng and Xiao, 2011). Wildlife have been recognized to play important roles in the ecology and transmission of *Cryptosporidium* spp., *G. duodenalis*, and *E. bieneusi* (Guo et al., 2021). The main route of transmission of these pathogens is the fecal–oral route, mainly through the consumption of contaminated food and water (Karanis et al., 2007; Ben Ayed et al., 2012; Galván et al., 2013). Infected humans might show typical symptoms of gastroenteritis, and immunocompromised individuals may experience severe symptoms and a higher risk of infection (Xiao and Fayer, 2008; Wang et al., 2013; Enserink et al., 2015). The similar distribution of these genotypes between humans and wild mammals indicates that there could be frequent cross-species transmission of these pathogens. The public health significance of these enteric protists depends on the distribution of genotypes and/or subtypes. Therefore, it is necessary to uncover the genetic characteristics of *Cryptosporidium* spp., *E. bieneusi* and *G. duodenalis* and elucidate their epidemiological features to implement effective therapeutic treatments.

Wild animals are often kept in captivity in zoological parks near urban areas and in close contact with humans, in particular animal keepers and visitors, thus serving as indicators of human risk of exposure to zoonotic agents in certain environments. In recent years, intestinal zoonotic pathogens have been found in captive wild animals in China (Li et al., 2016), which highlights that wild animals may be potential hosts for human infection with these infectious sources. However, there is little information about the prevalence and subtype distribution of these zoonotic pathogens in zoo animals in China. Hence, this study focused on the investigation of intestinal zoonotic protozoans in 12 species of wild animals in Qinghai-Tibet Plateau Wildlife Park, which is the highest altitude wildlife park in the world, to determine the genetic characteristics and subtype distribution of zoonotic pathogens.

## Materials and methods

### Fecal sample collection and DNA extraction

In this study, 167 and 103 fecal samples were collected from December 2021 to March 2022 and June 2022 to August 2022 from the Qinghai-Tibet Plateau Wild Zoo, respectively. In the zoos, an appropriate number of fecal samples were collected from each species screened, based on the number of individuals housed by species. In the late afternoon, the majority of the animals in the zoo were moved from their day to night enclosures. Fresh fecal samples were thus collected early in the morning before the cleaning of animal cages. For some avian species with no night enclosures, collection of stool samples was performed carefully directly on the ground or in nests. The collection of fecal samples was performed in the presence of zookeepers and was strictly controlled to minimize potential contamination between animal species. Each fresh sample was collected immediately after its defecation onto the ground, placed individually into a disposable plastic bag, transported to the laboratory and stored at -20°C prior to further analysis. After thawing the samples, 180–220 mg of each sample was placed in 2-mL microcentrifuge tubes for genomic DNA extraction using the QIAamp^®^ Fast DNA Stool Mini Kit (QIAGEN, Germany) according to the manufacturer’s specifications. Each DNA extraction product was stored at -20°C for further PCR amplification.

### PCR detection

In this study, PCRs were performed using the primers and annealing temperature (°C) described in **Table 1**, to amplify the *Cryptosporidium* sp. small subunit ribosomal RNA (SSU rRNA), *G. duodenalis* SSU rRNA, *E. bieneusi* sp. internal transcribed spacer (ITS), *Blastocystis* sp. SSU rRNA, and *Entamoeba* sp. 18S rRNA genes (Xiao et al., 1999; Verweij et al., 2003; Scicluna et al., 2006; Huber et al., 2007; Menounos et al., 2008; Feng and Xiao, 2011; Santín et al., 2011). The PCR of *Cryptosporidium* sp., *G. duodenalis* and *E. bieneusi* sp. were started at 95°C for 3 min followed by 35 cycles of 95°C for 30 s, corresponding annealing temperature (°C) in (**Table 1**) for 30 s, and 68°C for 60 s, with an extension at 68°C for 5 min. Each PCR of *Blastocystis* sp. consisted of 30 cycles of denaturation at 95°C for 30 s, corresponding annealing temperature (°C) in (**Table 1**) for 60 s, and extension at 68°C for 60 s; an initial denaturation step consisting of incubation at 95°C for 30 s and a final extension step consisting of incubation at 68°C for 5 min were also included. The 600-bp barcoding region of the SSU rRNA gene of *Entamoeba* sp. was amplified using the primers RD5 and BhRDr (Verweij et al., 2003). Each PCR consisted of 35 cycles of denaturation at 95°C for 30 s, annealing at 58.5°C for 60 s, and extension at 68°C for 60 s; an initial denaturation step consisting of incubation at 95°C for 3 min and a final extension step consisting of incubation at 68°C for 5 min were also included. The 10-μl PCR mixture contained 2 μl of DNA template, 0.5 μl of each forward and reverse primer (100 μM), 0.1 μl of Taq polymerase (0.5 U; New England BioLabs, USA), 0.2 μl of deoxyribonucleotide triphosphate (200 μM; New England BioLabs, USA), 1 μl of 10×ThermoPol Reaction Buffer (New England BioLabs, USA), and double-distilled water up to 10 μl. For positive controls, positive samples for *Blastocystis* sp. and *Entamoeba* sp. stored in the laboratory were used. No positive controls were available for other pathogens. Double-distilled water was used as a negative control.

**Table 1:** PCR primers for Five pathogens.

Pathogen	Target gene	Primer sequence(5’-3’)	Fragment (bp)	Annealing temperature (°C)	Reference
***Cryptosporidium* sp.**	SSU rRNA	Cry F: AACCTGGTTGATCCTGCCAGTAGTC	600	60	(Xiao et al., 1999)
		Cry R: TGATCCTTCTGCAGGTTCACCTACG			
	18S rRNA	Cry F1: TTCTAGAGCTAATACATGCG	1325	55	(Huber et al., 2007)
		Cry R1: CCCATTTCCTTCGAAACAGGA			
		Cry F2: GGAAGGGTTGTATTTATTAGATAAAG	819-825	59	
		Cry R2: CTCATAAGGTGCTGAAGGAGTA			
***Giardia duodenalis***	SSU rRNA	Gia F: AAGTGTGGTGCAGACGGACTC	497	60	
		Gia R: CTGCTGCCGTCCTTGGATGT			
	SSU rRNA	Gia F1: GACGCTCTCCCCAAGGAC	131	59	(Feng and Xiao, 2011)
		Gia R1: CTGCGTCACGCTGCTCG			
***Enteromicrosporidia bieneusi* sp.**	ITS	E F: GCTCTGAATATCTATGGTC	392	56	
		E R: ATCGCCGACGGATCCAAGTG			
***Blastocystis* sp.**	SSU rRNA	F: GAGCTTTTTAACTGCAACAA	600	58	(Scicluna et al., 2006)
		R: ATCTGGTTGATCCTGCCAGT			
		F1: GGAGGTAGTGACAATAAATC	500	56	(Santín et al., 2011)
		R1: TAAGACTACGAGGGTATCTA			
		F2: CGAATGGCTCATTATATCAGTT	260	56	(Menounos et al., 2008)
		R2: TCTTCGTTACCCGTTACTGC			
***Entamoeba* sp.**	18S rRNA	RD5: GTTGATCCTGCCAGTATTATATG	550	58.5	(Verweij et al., 2003)
		BhRDr: CACTATTGGAGCTGGAATTAC			

### Sequencing and phylogenetic analysis

The PCR product of the positive samples was purified using the EasyPure^®^ Quick Gel Extraction Kit (TransGen, China) and cloned into *E. coli* DH5α using the PmdTM 19-T Vector Cloning Kit (TaKaRa, Japan). At least two positive clones were sequenced by AuGCT Biotech (Shanxi, China). The obtained sequences were confirmed by a BLASTn search in GenBank. Phylogenetic trees were constructed from the aligned sequences using the neighbor-joining (NJ) method in MEGA7 (http://www.megasoftware.net/) with 500 replicates to assess the robustness of clusters.

### Statistical analysis

The 95% confidence intervals were calculated using the OpenEpi program (https://www.openepi.com/Proportion/Proportion.htm, accessed on 15 November 2020).

## Results

*Entamoeba* sp. infection was observed in 21 samples, including from 2 white-lipped deer, 8 Sika deer, 6 blue sheep, 2 wolves, and 3 bears, with an overall infection rate of 12.6% (21/167). The prevalence of *Entamoeba* sp. in *Cervidae* was 40.9% (10/22, 95% CI 20.4–61.5), which was higher than that in *Canidae* 28.6% (2/7, 95% CI (4.9–62.0), *Felidae* 20.0% (2/10, 95% CI 4.8–44.8), *Ursidae* 12.5% (3/24, 95% CI 0.7–25.7), and *Bovidae* 12.0% (9/75, 95% CI (4.6–19.4) (**Table 2**). Molecular diagnosis of 103 stool samples revealed *Entamoeba* sp. infection in 5 samples, including from 1 snow leopard, 1 tiger, 1 Tibetan argali and 2 mouflon. The prevalence of *Entamoeba* sp. in *Felidae* was 20.0% (2/10, 95% CI 4.8–44.8) higher than that in *Bovidae* 8.1% (3/37, 95% CI (0.7–16.9). Moreover, 1 white-lipped deer and 1 bear were positive for *Blastocystis* sp. in winter and summer, respectively. No positive DNA was detected in lion, peacock and bar-headed goose samples.

**Table 2:** The total number of animal stool samples collected for this study and the percentage of positive samples obtained by the PCR method.

Animals	*Blastocystis*				*Entamoeba*			
	Winter		Summer		Winter		Summer	
	Tested	Positive (%, 95% CI)	Tested	Positive (%, 95% CI)	Tested	Positive (%, 95% CI)	Tested	Positive (%, 95% CI)
Feline								
Snow leopard	6	0	3	0	6	0	3	1 (33.3%, 20.0-86.7)
Tiger	24	0	4	0	24	0	4	1 (25.0%, 17.4-67.4)
Lion	26	0	3	0	26	0	3	0
Total	56	0	10	0	56	0	10	2 (20.0%, 4.8-44.8)
Cervidae								
White-lipped Deer	8	1 (12.5%, 10.4-35.4)	11	0	8	2 (25.0%, 5.0-55.0)	11	0
Sika deer	14	0	11	0	14	8 (57.1%, 31.2-83.1)	11	0
Total	22	1 (4.5%, 4.2-13.2)	22	0	22	10(40.9%,20.4-61.5)	22	0
Bovidae								
Blue sheep	15	0	7	0	15	6 (40.0%, 15.2-64.8)	7	0
Tibetan argali	11	0	10	0	11	0	10	1 (10.0%, 8.6-28.6)
mouflon	12	0	20	0	12	0	20	2 (10.0%, 3.1-23.1)
Total	38	0	37	0	38	6 (15.8%, 4.2-27.4)	37	3 (8.1%, 0.7-16.9)
Canidae								
Wolf	7	0	4	0	7	2 (28.6%, 4.9-62.0)	4	0
Total	7	0	4	0	7	2 (28.6%, 4.9-62.0)	4	0
Ursidae								
Bear	24	0	4	1 (25.0%, 17.4-67.4)	24	3 (12.5%, 0.7-25.7)	4	0
Total	24	0	4	1 (25.0%, 17.4-67.4)	24	3 (12.5%, 0.7-25.7)	4	0
Pheasants								
Peacock	10	0	14	0	10	0	14	0
Total	10	0	14	0	10	0	14	0
Ducks								
Bar-headed Goose	10	0	12	0	10	0	12	0
Total	10	0	12	0	10	0	12	0
Total	167	1 (0.6%, 0.6-1.8)	103	1 (1.0%, 0.9-2.9)	167	21 (12.6%, 7.5-17.6)	103	5 (4.9%, 0.7-9.0)

**Figure 1:**
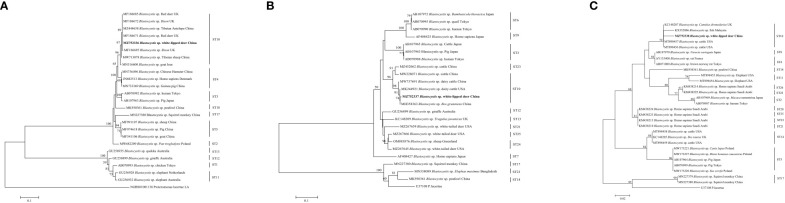
Phylogenetic tree based on the partial SSU rRNA sequences of representative *Blastocystis* members constructed using the neighbor-joining method. New sequences are indicated in bold text. The scale bar represents 0.1 **(A, B)**, 0.02 **(C)** substitutions per nucleotide.

**Figure 2:**
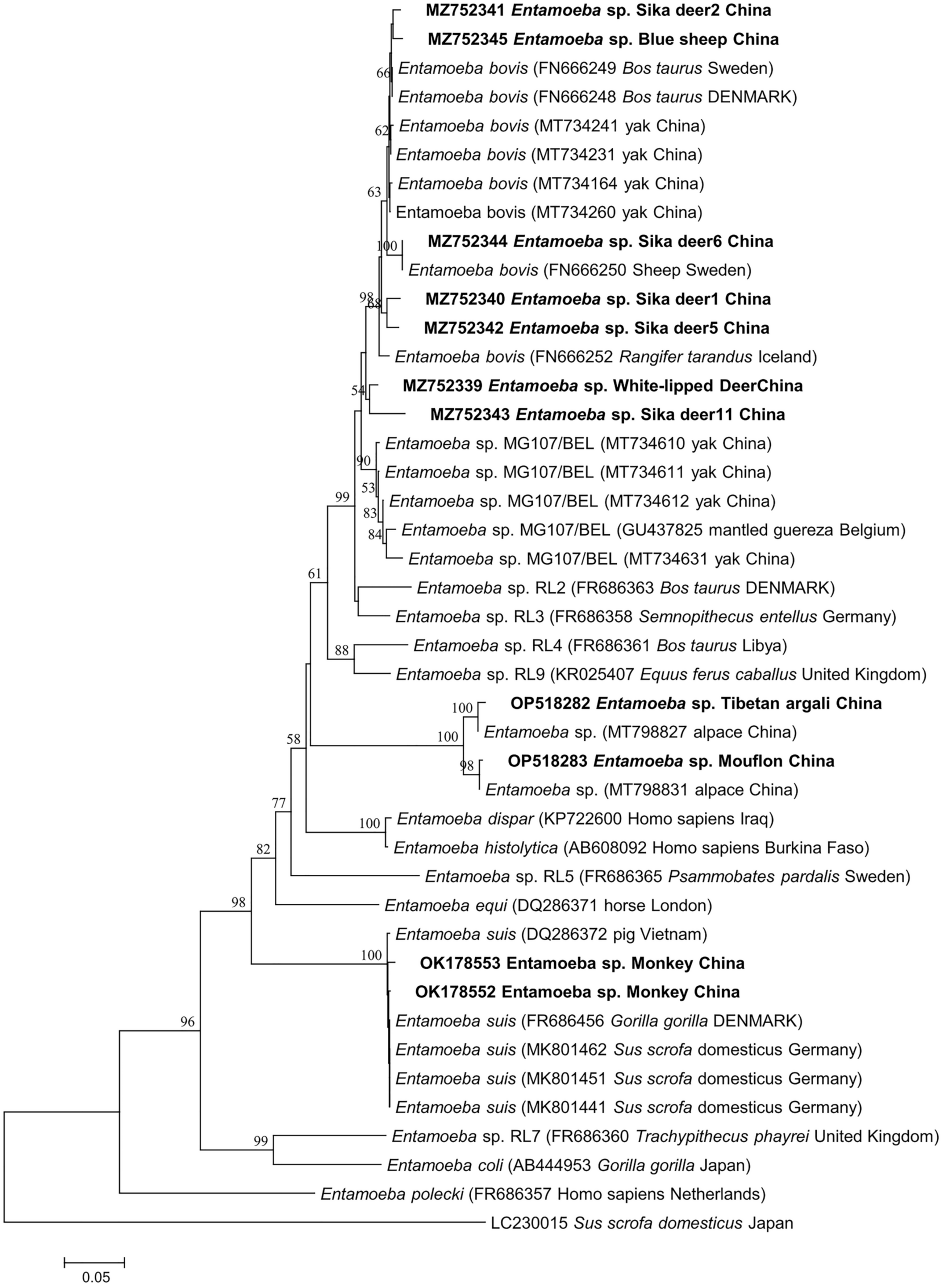
Phylogenetic tree based on the partial 18S rRNA sequences of representative *Entamoeba* members constructed using the neighbor-joining method. New sequences are indicated in bold text. The scale bar represents 0.05 substitutions per nucleotide.

This study used three species or genus-specific primers of *Blastocystis* to identified that it was ST10 animal-specific STs subtype of *Blastocystis*. The three pairs of primers sequenced three different *Blastocystis* sequences, which were approximately 500 bp (A), 600 bp (B) and 260 bp (C) in length (**Figure 1**), it clearly improves the accuracy required for subtyping. Newly acquired sequences belong to ST10, ST10 formed a clade with sequences from Red deer, *Bison*, Tibetan Antelope, Tibetan sheep, and goat (**Figure 1A**). ST10 along with sequences isolated from cattle, dairy cattle, and *Bos grunniens* clustered together (**Figure 1B**). ST10 along with sequences originating from *Camelus dromedarius*, fish and cattle clustered together (**Figure 1C**). A total of 11 representative sequences were obtained from 26 *Entamoeba* sp. isolates in the present study. The final sequences were deposited in NCBI GenBank under the accession numbers listed in **Table 3**. The sequences obtained in this study showed high identity with the reference sequences of *Entamoeba* sp. in GenBank. Newly acquired sequences belonged to *Entamoeba bovis* and *Entamoeba suis*. Phylogenetic analysis of the sequences obtained in this study was based on the neighbor-joining method. Sequences MZ752339–MZ752345 along with sequences originating from *Bos taurus*, yak, sheep and *Rangifer tarandus* clustered together. Sequences OP518282 and OP518283 grouped together with sequences mainly from alpaca. Sequences OK178552 and OK178553 clustered together with sequences from pig, *Gorilla* and *Sus scrofa domesticus* (**Figure 2**). This is the first time that *E. bovis* and *E. suis* were detected in the QTPA, China.

**Table 3:** Accession numbers of DNA sequences from this study deposited in GenBank.

Obtained sequences					The closest BLASTn match	
Pathogens	Animal species	Target genes	Accession numbers	Length (bp)	Identities (%)	Accession numbers (host, country)
*Entamoeba* sp.	White-lipped Deer	18S rRNA	MZ752339	584	97.61	MT734612 yak China
	Sika deer		MZ752340	584	97.07	MT734164 yak China
	Sika deer		MZ752341	583	98.63	FN666249 *bos taurus* Sweden
	Sika deer		MZ752342	583	96.92	FN666252 *rangifer tarandus* Iceland
	Sika deer		MZ752343	584	99.83	FN666252 *rangifer tarandus* Iceland
	Sika deer		MZ752344	584	94.53	FN666250 *aries ovis* Sweden
	Blue sheep		MZ752345	584	98.63	FN666249 *bos taurus* Sweden
	Monkey		OK178552	550	100.00	MK801431 *susscrofa demesticus* Germany
	Monkey		OK178553	550	99.27	FR686456 gorilla United Kingdom
	Tibetan argali		OP518282	558	99.10	MT798827 alpaca China
	Mouflon		OP518283	580	99.47	MT798831 alpaca China
*Blastocystis* sp.	White-lipped Deer	SSU rRNA	MZ752336	610	100.00	MZ444658 Tibetan Antelope China
	White-lipped Deer		MZ752337	548	99.04	MH358363 *bos grunniens* China
	White-lipped Deer		MZ752338	267	99.63	MG831506 cattle Malaysia

The identity analysis of the 18S rRNA gene revealed that two sequences of *E. suis* isolates identified in monkeys were identical to those from Vietnamese pig in Vietnam (DQ286372). Similarly, the sequence OP518282 from Tibetan argali showed 95.6% identity with the GenBank sequence MT798827 (from alpaca in China). In one mouflon-derived *Entamoeba* sp. isolate, the sequence had 99.5% identity with that from alpaca in China (MT798831). The MZ752339 and MZ752345 sequences had identities (25.0% and 99.67%) related to sequences from Rangifer tarandus in Iceland (FR666252) and yak in China (MT734231), respectively. Moreover, one sika deer-derived *E. bovis* sequence had 99.7% identity with a sequence from a sheep in Sweden (FR666250). The identity analysis of the SSU rRNA gene revealed that one sequences of ST10 isolates identified in white-lipped deer was identical to those from Tibetan Antelope in the China (MZ444658) (**Figure 1A**). One white-lipped deer ST10 sequences had 93.70% identity with that from an *Bos grunniens* in China (MH358363) (**Figure 1B**). In terms of one white-lipped deer ST10 isolates, the sequences had 97.80% identity with that from cattle in USA (MT898456) (**Figure 1C**).

## Discussion

The wild animals in zoos are closely related to people’s lives, especially those in free-range or semifree-range zoos. Keepers and veterinarians, as well as tourists, come into close contact with these wild animals, resulting in a high probability of zoonotic diseases. Therefore, it is necessary to investigate the parasites of wild animals in wildlife parks. Due to the lack of data on parasitic pathogens infecting wildlife, this study investigated the presence of infectious sources in Qinghai-Tibet Plateau Wildlife Park. In the winter, *Blastocystis* and *Entamoeba* were detected in the present study in white-lipped deer, Sika deer, blue sheep, wolf and bear. *Blastocystis* and *Entamoeba* were also detected in the present study in bear, snow leopard, tiger, Tibetan argali and mouflon in the summer. Significantly, none of the animals exhibited diarrheal episodes or other obvious gastrointestinal symptoms as confirmed by zookeepers and licensed veterinarians.

*Blastocystis* is a common intestinal protozoan parasite, and its pathogenicity is still uncertain. It is believed that zoonotic STs are usually transmitted between animals and humans, as some animal derived STs are huge potential hosts of human infections (Li et al., 2019; Zhu et al., 2020). Previous studies have surveyed the prevalence and subtypes of *Blastocystis* in animals from Hangzhou, Dalian, and Suzhou zoos in China, and positive fecal samples were detected in red deer and *Pavo critatus*, with a positive rate of 6.0% (Li et al., 2020b). *Blastocystis* was also isolated from sika deer in a zoo in Southwest China (Deng et al., 2021). In a Danish study, an elk was found harboring ST10 (Stensvold et al., 2009). The present study found that (4.5%) 1/22 *Cervidae* samples contained *Blastocystis*. Two previous studies investigated the presence of *Blastocystis* in six different deer species and neither was able to identify *Blastocystis* in any of the samples (Abe et al., 2002; Lim et al., 2008). The prevalence of *Blastocystis* sp. in zoo animals examined in this study was 0.6% (1/167) and 1.0 (1/103) in winter and summer, respectively, which was lower than that in captive wild animals in Qinling, China (40.2%, 200/497) (Zhao et al., 2017), in zoo animals in Japan (39.0%, 46/118) (Abe et al., 2002), and was also lower than that in zoo animals in three cities in China (6.0%, 27/450) (Li et al., 2020b). However, it is difficult to explain the differences in *Blastocystis*. The prevalence rate between different countries or within the same country is influenced by many factors, such as sample size, animal species, or management methods. In the present study, ST10 was identified in 1 *Blastocystis* sp.-positive samples from captive wildlife. The majority of STs (ST1-7, ST10, ST13-15, and ST17) have been identified in Artiodactyla to date (Alfellani et al., 2013; Wawrzyniak et al., 2013). Among them, ST10 was the most common subtype in cattle in the China (Zhu et al., 2017). The distribution of STs in white-lipped deer in the present study was consistent with a previous study in the Qinglin Mountains in China, in which all isolates identified belonged to ST10 (Zhao et al., 2017). Overall, these data suggest that deer may serve as natural hosts of *Blastocystis* sp.

Infection with zoonotic parasites of *Entamoeba* is common in most domestic animals and represents a serious threat to human health. Evidence has shown that *Entamoeba* is indeed transmissible between nonhuman primates and humans (Levecke et al., 2015). The QTPR is a high-altitude region in China with unique climatic and environmental features. Although studies have investigated *Entamoeba* infection in yaks in Qinghai Province (Ren et al., 2021), the overall situation of *Entamoeba* infections in zoo animals in the QTPR is unknown, and the potential risk of human exposure to *Entamoeba* infection is difficult to evaluate. Here we conducted an investigation into *Entamoeba* sp. infection in the Qinghai-Tibet Plateau Wildlife Park. The prevalence of *Entamoeba* sp. in zoo animals examined in this study was 12.6% (21/167) and 4.9% (5/103) in winter and summer, respectively, which were lower than those in cattle farms in Japan (72.0%, 18/25) (Matsubayashi et al., 2018). *Entamoeba* sp. has also previously been identified in captive nonhuman primates of 24 zoological gardens in China (Li et al., 2015). Considering that the prevalence of intestinal parasites is usually related to many factors, including detection methods, living environment, host health status, age, and sampling size, further research is needed to reveal the risk factors of *Entamoeba* infection in animals. The pathogenicity of *E. bovis* has not been well documented thus far. In our survey, none of the animals exhibited clinical symptoms, such as diarrhea. Therefore, it is possible that *E. bovis* and *E. suis* were less pathogenic, and the infection persists for a long time without being cured. While *Entamoeba* and *Blastocystis* infections in zoo animals have been reported in China, no reports have been found for the Qinghai Tibet Plateau. Particular attention should be given to their presence and the potential risk of contact-mediated transmission. It is not clear at present whether the lower rate of *Blastocystis* infection detected in wild animals is an artifact due to this group being relatively under sampled or is real, and only further sampling from wild animal populations can answer this question. This epidemiological survey provides the necessary information for taking preventive and control measures that should help to reduce the burden of *Blastocystis* sp. and *Entamoeba* sp. in zoos and the risks for zoonotic transmission to animal handlers.

*Cryptosporidium* was detected in camels in the Anhui Zoo (Gu et al., 2016), and it was also detected in fecal samples from black leopard (*Panthera pardus*), black-necked crane (*Grus nigricollis*), and white-eared pheasant (Karanis et al., 2007). *Giardia duodenalis* is common in wild and captive nonhuman primates (Beck et al., 2011; Karim et al., 2015). *Giardia* was detected in the feces of cats and snow leopards in the Zhengzhou Zoo, and *E. bieneusi* sp. was detected in the feces of cats, alpacas, lions, tigers, and peacocks (Li et al., 2015). The prevalence of *E. bieneusi* was 19.75% (80/405) and 27.4% (29/106) in the Yunnan and southwestern regions, respectively (Deng et al., 2017; Wu et al., 2018). However, current research has failed to detect these three pathogens. The observed differences in the prevalence of these parasites among different zoo animals may be due to variations in food density, geography, management systems, sample sizes and climate; however, the possible influence of these factors on prevalence remains entirely unexplored, and further studies are still necessary to elucidate this aspect.

Two protozoan parasites, *Blastocystis* and *Entamoeba*, were detected in the current study, suggesting the zoonotic potential of *Blastocystis* sp. and *Entamoeba* sp. in the Qinghai-Tibet Plateau Wildlife Park and highlighting their potential threat to human health. We found no effect on season on *Blastocystis* sp. and *Entamoeba* sp. colonization. However, our study has some limitations, including the small number of samples and lack of morphological observation and of assessment of clinical symptoms. In future studies, we will apply a wider range of molecular biological detection methods to analyze parasitic infections in zoo and wild animals in the Qinghai Tibet Plateau to provide a reference for future research.

## Data availability statement

The datasets presented in this study can be found in online repositories. The names of the repository/repositories and accession number(s) can be found in the article/supplementary material.

## Ethics statement

The animal study was reviewed and approved by the Ethics Committee of Qinghai University (protocol code: SL-2021016, date of approval: 2021.3.16).

## Author contributions

TQ: Collection of animal samples, Data curation, Formal analysis, Investigation, Writing-review and editing. WZ: Investigation. YS: Writing-review and editing. LG: Investigation. JL: Writing-review and editing. MK: Conceptualization, Funding acquisition, Resources, Writing original draft, Writing-review and editing. All authors contributed to the article and approved the submitted version.
